# Gut Colonisation and Multidrug-Resistant Urinary Tract Infections in Hospitalised Kidney Transplant Recipients: A Single-Centre Retrospective Study

**DOI:** 10.3390/antibiotics15070656

**Published:** 2026-07-01

**Authors:** Laura Loiacono, Assunta Navarra, Claudia Rotondo, Valentina Dimartino, Fabio Iacomi, Amina Abdeddaim, Raffaella Lionetti, Paolo De Paolis, Carla Fontana, Elisa Biliotti, Gianpiero D’Offizi

**Affiliations:** 1 Clinical and Research Infectious Diseases Department, National Institute for Infectious Diseases “Lazzaro Spallanzani”, IRCCS, 00149 Rome, Italy; laura.loiacono@inmi.it (L.L.); fabio.iacomi@inmi.it (F.I.); amina.abdeddaim@inmi.it (A.A.); raffaella.lionetti@inmi.it (R.L.); elisa.biliotti@inmi.it (E.B.); gianpiero.doffizi@inmi.it (G.D.); 2 Epidemiology Unit, National Institute for Infectious Diseases “Lazzaro Spallanzani”, IRCCS, 00149 Rome, Italy; assunta.navarra@inmi.it; 3 Microbiology and Biobank Unit, National Institute for Infectious Diseases “Lazzaro Spallanzani”, IRCCS, 00149 Rome, Italy; valentina.dimartino@inmi.it (V.D.); carla.fontana@inmi.it (C.F.); 4 Nephrology, Dialysis and Transplantation Unit, San Camillo-Forlanini Hospital, 00149 Rome, Italy; pdepaolis3@alice.it

**Keywords:** kidney transplantation, complicated urinary tract infection, multidrug-resistant organisms, carbapenemase-producing *Klebsiella pneumoniae*, rectal colonisation, carbapenem-resistant *Enterobacterales*

## Abstract

**Background/Objectives**: Urinary tract infections (UTIs) represent the most common infectious complication following kidney transplantation. An increasing number of UTIs are caused by multidrug-resistant organisms (MDROs). The role of intestinal MDRO colonisation in complicated urinary tract infections (cUTIs) among kidney transplant recipients is not fully understood. **Methods**: We conducted a retrospective, single-centre study of kidney or kidney–pancreas transplant recipients hospitalised for infectious diseases. Each hospitalisation was analysed as a separate event. Routine rectal screening targeted carbapenem-resistant *Enterobacterales* and vancomycin-resistant/vancomycin-variable enterococci. **Results**: The study included 65 hospitalisations from 52 kidney transplant recipients, with some patients contributing multiple admissions. cUTIs accounted for 63.1% of admissions, and 22.0% of cUTIs were associated with concomitant bloodstream infection (BSI). The most frequently isolated pathogens were *Klebsiella pneumoniae* (58.8%) and *Escherichia coli* (41.2%). Extended-spectrum β-lactamase (ESBL) production was detected in 50% of *E. coli* isolates, while carbapenemase production was identified in 60% of *K. pneumoniae* isolates. MDRO rectal carriage was detected in 43.1% of cases and was more frequent in cUTI than in other infections (53.7% vs. 25.0%, *p* = 0.024). Carbapenemase-producing *K. pneumoniae* (CP-KP) rectal carriage was also more frequent in cUTI (31.7% vs. 4.2%, *p* = 0.011), but did not remain statistically significant after adjustment for urinary stent presence (odds ratio 7.1, 95% CI 0.7–66.2; *p* = 0.087). Nevertheless, CP-KP rectal carriage was associated with CP-KP cUTI aetiology (PPV 75.0%; NPV 86.4%). The median length of hospital stay (LoS) was 15 days. In multivariable analysis, a longer median LoS was associated with BSI (12.2 days; 95% CI: 0.8–23.6; *p* = 0.037), urinary stent presence (6.8 days; 95% CI: 1.5–12.2; *p* = 0.014), and older age (2.3 days; 95% CI: 0.7–4.0; *p* = 0.007). **Conclusions**: Rectal CP-KP colonisation may represent a potential marker of cUTI risk, although its independent association was not confirmed after adjustment. These findings should be interpreted with caution, given the study design and sample size and require confirmation in larger prospective studies. Rectal screening may contribute to early risk stratification, whereas its role in guiding empirical therapy remains to be prospectively evaluated.

## 1. Introduction

Urinary tract infections (UTIs) are the most common infectious complication in kidney transplant (KT) recipients, significantly impacting hospitalisation, morbidity, mortality, graft function and long-term graft survival [1,2]. The incidence of UTIs in KT recipients could vary widely from 7% to 80%, with an estimated pooled incidence of approximately 33.5%, and recurrence rates could reach up to 36% [3,4,5]. This variability reflects differences in study design, diagnostic criteria, antimicrobial prophylaxis strategies and follow-up duration. UTIs are the leading cause of bloodstream infections (BSIs) in KT recipients, accounting for at least 40% of cases [2,6]. Although UTIs can occur at any time after transplantation, they are most prevalent in the first three to six months after KT. This period is characterised by intense immunosuppression and frequent urological interventions [7,8]. The predominant causative agents are Gram-negative bacteria, with *Escherichia coli*, *Klebsiella* spp., *Pseudomonas aeruginosa* and *Proteus mirabilis* accounting for most episodes. Gram-positive pathogens, such as *Enterococcus* spp. and *Staphylococcus aureus*, play a less prominent role.

Over the past few years, the epidemiology of UTIs in KT recipients has been significantly influenced by the growing prevalence of multidrug-resistant organisms (MDROs), particularly extended-spectrum β-lactamases (ESBLs)-producing *Enterobacterales*, carbapenem-resistant *Enterobacterales* (CREs) and vancomycin-resistant/vancomycin-variable enterococci (VRE/VVE) [9,10]. These pathogens complicate treatment choices, limiting options and are associated with worse clinical outcomes, prolonged hospitalisation and a higher risk of systemic infection.

Post-transplant UTIs are the result of several factors and reflect the interaction between host-related features, pathogen characteristics and anatomical or procedural determinants [1]. Established risk factors include female sex, older age, diabetes, hypertension, urinary tract abnormalities, indwelling urinary catheters or ureteral stents, acute rejection episodes and a history of recurrent UTIs before transplantation [2,8,10,11]. Meanwhile, the role of immunosuppressive therapy remains controversial [12], but higher maintenance of corticosteroid doses has been associated with an increased risk of infection [1,12].

Among emerging risk factors, intestinal colonisation by MDROs has gained increasing attention. Many transplant centres routinely perform active surveillance for MDRO gut colonisation, particularly for VRE, ESBL-producing *Enterobacterales* and CRE, although the clinical implications remain controversial. Current international guidelines recommend systematic screening, primarily in liver transplant recipients and in settings with a high epidemiological burden of multidrug-resistant Gram-negative bacteria, while its role in other solid organ transplant populations, including KT recipients, is less clearly defined and often guided by local epidemiology [13,14].

Among MDROs, carbapenemase-producing *Klebsiella pneumoniae* (CP-KP) is of particular concern in transplant populations, given its high propensity for invasive infection, its association with limited therapeutic options, and its documented impact on morbidity and mortality [13,15].

In high-burden settings, CP-KP has emerged as a leading pathogen in severe infections among solid organ transplant recipients [13,16].

MDRO intestinal colonisation has been associated with an increased risk of subsequent infection; however, its predictive value for the aetiological diagnosis and clinical course of complicated urinary tract infections (cUTIs) in KT recipients remains poorly understood.

In particular, there is limited data on whether colonisation status could be usefully employed in the early stages to inform risk stratification, guide empirical antimicrobial therapy, or predict adverse outcomes such as BSI or prolonged hospitalisation. However, the available evidence remains heterogeneous, and the predictive value of intestinal colonisation for subsequent infection, particularly for defining aetiology and clinical outcomes, has been inconsistently reported across studies.

Therefore, we performed a retrospective observational study of KT recipients hospitalised for infectious diseases to (i) estimate the prevalence of UTIs and identify associated risk factors, (ii) characterise the burden of MDROs among pathogens causing cUTIs, and (iii) assess the association between rectal MDRO colonisation and cUTI aetiology, with a particular focus on CP-KP. We also examined clinical outcomes, including concomitant BSI and the length of hospital stay (LoS), to explore the potential utility of rectal screening as a real-time tool to support early risk stratification and guide empirical antimicrobial therapy in hospitalised KT recipients.

## 2. Results

### 2.1. Study Population and Baseline Characteristics

Between June 2023 and January 2025, a total of 65 hospitalisations due to infectious diseases were recorded among kidney or kidney/pancreas transplant patients. A total of 52 KT patients were included in the study: seven experienced two hospitalisations, and three were hospitalised three times during the study period. The baseline characteristics of the enrolled patients are reported in Table 1. The latter provides a comprehensive overview of both clinical characteristics and microbiological findings. Most admissions involved male patients (56.9%), with a median age of 57 years (interquartile range (IQR) 48–67). According to the “Kidney Disease: Improving Global Outcomes” (KDIGO) classification [17], 84.6% of patients had moderate-to-severe kidney disease (estimated glomerular filtration rate (eGFR) < 60 mL/min/1.73 m^2^), with a median eGFR of 30 mL/min/1.73 m^2^ (IQR 18–47). A urinary stent was present at admission in 36.9% of cases.

Trimethoprim-sulfamethoxazole prophylaxis was conducted in 32% (21/65) of hospital admissions.

A substantial number of events occurred during the early post-transplant period when patients are clinically vulnerable, as indicated by the fact that the time since transplantation was less than one year in 30.8% of cases. All cases involved calcineurin inhibitors, which were frequently combined with corticosteroids (95.4%) and mycophenolate mofetil (72.3%). Among those receiving steroids, 35.5% received a dose of ≥10 mg/day. This background reflects an intensively immunosuppressed population, which is consistent with hospitalisations related to infectious diseases in transplant recipients.

Because some patients contributed more than one hospitalisation, observations were not fully independent and should be interpreted accordingly.

### 2.2. Admissions Related to Infection and the Burden of Complicated Urinary Tract Infections (cUTIs)

Among the infectious diseases requiring hospitalisation, the prevalence of cUTIs was 63.1% (41/65 admissions), and 22% (9/41 admissions) of cUTIs had a concomitant BSI (the detailed description in Supplementary Table S1). Recurrent UTIs occurred in 15.4% of patients. Other types of infections were: pneumonia (14/24, 58.3%), abdominal infections (4/24, 16.7%), sepsis (3/24, 12.5%), herpes zoster (2/24, 8.3%) and CMV infection (1/24, 4.2%). Patients with cUTI more frequently had a urinary stent (48.8% vs. 16.7%, *p* = 0.016) and were more commonly receiving an immunosuppressive regimen that included corticosteroids (100% vs. 87.5%, *p* = 0.046), irrespective of dosage, compared with patients hospitalised for other types of infections. The prevalence of MDRO rectal carriage was 43.1% in the overall cohort; CP-KP and VRE/VVE *faecium* were detected in 21.5% and 24.6% of cases, respectively, while VRE *faecalis* was detected in one case. The prevalence of MDRO rectal carriage was significantly higher among patients with cUTI compared to those with other infections (53.7% vs. 25.0%, *p* = 0.024). The prevalence of VRE/VVE rectal colonisation did not differ substantially between the two groups (29.3% vs. 16.7%, *p* = 0.373), while colonisation with CP-KP was markedly more frequent in patients with cUTI (31.7% vs. 4.2%, *p* = 0.011).

Table 1 provides a comprehensive overview of both clinical and microbiological characteristics, while key findings are summarised in the text to facilitate interpretation.

Although these differences were statistically significant in univariable comparisons, their role as independent factors was not confirmed in multivariable analysis (see Section 2.3).

### 2.3. Multivariable Analysis of Factors Associated with Complicated Urinary Tract Infections (cUTIs)

In exploring predictors of cUTI, corticosteroid-containing immunosuppressive therapy was excluded from the candidate predictors due to its very limited variability, as only three patients did not receive corticosteroids. Stepwise logistic regression identified urinary stent presence and CP-KP rectal colonisation as the variables retained in the final multivariable model. Both predictors showed large effect estimates, although neither reached conventional statistical significance (urinary stent: odds ratio [OR] 3.3, 95% confidence interval [CI]: 0.8–13.1, *p* = 0.094; CP-KP rectal colonisation: OR 7.1, 95% CI: 0.7–66.2, *p* = 0.087). The wide CIs, particularly for CP-KP rectal colonisation, indicate substantial uncertainty around the effect estimates, likely reflecting the limited number of events. In accordance with the modelling strategy described in the Methods section, the number of predictors was limited to two, based on the 24 observations in the smaller outcome category. Among all candidate two-predictor models, comparison using the Bayesian Information Criterion (BIC) confirmed that the stepwise-selected model, including urinary stent presence and CP-KP rectal colonisation, yielded the lowest BIC. However, the presence of multiple competing models with similar support (ΔBIC < 6) suggests a non-negligible degree of model-selection uncertainty.

Overall, these findings suggest that both factors may be associated with cUTI; however, independent effects could not be confirmed due to the limited precision of the estimates. Larger studies are needed to confirm these observations and to better delineate the relative contribution of each predictor.

Out of 41 hospital admissions for cUTI, the aetiological agent was identified in 34 cases (82.9%). *K. pneumoniae* and *E. coli* were the most frequently isolated pathogens, accounting for 58.8% (20/34) and 41.2% (14/34) of cases, respectively. *Enterococcus* spp. was identified as the causative agent in only two episodes of cUTI. Antibiotic resistance analysis showed that 50% of *E. coli* isolates produced ESBL, while carbapenemase production was detected in 60% of *K. pneumoniae* isolates. The carbapenemase types identified included nine *bla*_KPC_, two *bla*_NDM_ and one *bla*_OXA-48_ (Table S1). Analysis of the relationship between rectal colonisation and UTIs due to the same organism revealed an association for CP-KP, with a negative predictive value (NPV) of 86.4% (95% CI 65.1–97.1%) and a positive predictive value (PPV) of 75.0% (95% CI 42.8–94.5%) (Table 2), whereas no association was observed for VRE/VVE colonisation. These estimates should be interpreted with caution given the limited number of cases and the resulting wide confidence intervals, which limit the precision of these findings.

Among admissions with cUTI, the overall median LoS was 15 days (IQR 10–20). It should be noted that other clinically relevant factors potentially influencing LoS, such as infection severity at admission, Intensive Care Unit (ICU) admission, graft dysfunction, timing of source control, and antimicrobial therapy, were not included in the analysis due to incomplete data availability and sample size constraints.

The main baseline factor associated with a longer LoS was the presence of BSI (median increase of 14.0 days; 95% CI: 5.7 to 22.3; *p* = 0.002). BSIs were mostly caused by CP-KP (4/9, 44.4%) and *E. coli* (3/9, 33.3%). In the 5 cases with an identified cUTI aetiology, the concordance between BSIs and urinary isolates was 80%. Although not reaching statistical significance, LoS was also longer in the presence of a urinary stent (median increase of 7.0 days; 95% CI −1.5 to 15.5; *p* = 0.104), and with increasing age (for each 10-year increment, the median LoS increased by 2 days, 95% CI −0.5 to 4.5; *p* = 0.112). In multivariable analysis (Table 3), a longer median LoS remained significantly associated with BSI (12.2 days, 95% CI: 0.8–23.6; *p* = 0.037), as well as with urinary stent presence (6.8 days, 95% CI 1.5–12.2; *p* = 0.014) and with older age (2.3 days; 95% CI: 0.7–4.0; *p* = 0.007).

## 3. Discussion

UTIs remain the most frequent infectious complication after kidney transplantation, representing a major driver of hospitalisation and downstream morbidity, contributing to BSI, graft dysfunction and increased healthcare utilisation [2,3,6,18,19]. In our cohort of KT recipients hospitalised for infectious diseases, cUTIs accounted for 63.1% of admissions. Complications arising from BSI occurred in 22% of cases, which confirms the central role of the urinary tract as a source of systemic infection in this population. Furthermore, UTIs recurred in 15.4% of patients, consistent with the recurrence rates observed in diverse KT cohorts [20,21,22].

From an aetiological perspective, Gram-negative bacteria predominate; among cUTI admissions with an identified pathogen (82.9%), *K. pneumoniae* and *E. coli* accounted for 58.8% and 41.2% of episodes, respectively, while *Enterococcus* spp. was uncommon in line with previous studies [8,23]. The antimicrobial resistance profile observed in our cohort is particularly concerning: ESBL production was detected in 50% of *E. coli* isolates, while carbapenemase production was identified in 60% of *K. pneumoniae* isolates. While rates of ESBL production among uropathogenic *E. coli* in KT recipients are frequently reported around 40% in Europe, with substantial heterogeneity across settings [1,24], the burden of carbapenem resistance in *K. pneumoniae* has shown a clear worsening trend in recent years, particularly in higher-burden countries and tertiary care networks.

Recent European surveillance data provide a robust context for these findings. Data on carbapenem resistance among invasive *K. pneumoniae* isolates in the EU/EEA show wide variability between countries (ranging from 0% to 69.7% in 2023), together with an overall increasing trend, with the population-weighted mean rising from 10.4% in 2019 to 13.3% in 2023 [25]. Although these reports are not specific to UTIs, they reflect the broader hospital ecology and the growing circulation of high-risk CR-KP clones. In addition, a recent systematic review of hospital-acquired *K. pneumoniae* infections estimated a global prevalence of carbapenem-resistant strains of 28.69%, with marked geographical variability [26]. Taken together, these observations suggest that the high prevalence of CR-KP observed in our cohort is more likely to reflect the epidemiological setting of a high-burden referral centre rather than an isolated finding. This evidence should also be interpreted in light of the characteristics of our centre, which functions as a referral hospital for infectious diseases and manages a high proportion of clinically complex and previously exposed patients, potentially contributing to a higher burden of antimicrobial resistance. In this context, our results may still provide meaningful real-world insights into clinical scenarios in which antimicrobial resistance is a major concern.

Importantly, evidence from transplant-specific cohorts supports this interpretation. In KT recipients, infections caused by CRE, particularly CR-KP, have consistently been linked to worse clinical outcomes, including increased mortality, graft-related complications, and prolonged hospitalisation [9,15,27,28,29,30,31]. In addition, cohort studies in transplant populations have shown that MDRO colonisation is not only frequent but also clinically relevant, as it significantly increases the risk of subsequent infection and impacts the post-transplant clinical course [32].

Overall, the available literature indicates that in KT recipients, early infections due to CR-KP are associated with a poorer prognosis, especially in more clinically complex cases or in patients requiring intensive care or invasive support [9,15]. These findings are in line with broader transplantation data showing that infections caused by CRE are associated with high attributable mortality, particularly when there is a delay in initiating effective antimicrobial therapy [21,27,28,29,30,31].

More recently, multi-centre data suggest that, in the era of improved access to effective agents and specialised management pathways, solid organ transplant recipients (SOTRs) with CRE do not necessarily experience worse short-term mortality than matched non-transplant patients, highlighting the importance of early recognition and appropriate treatment strategies [9,16].

The novelty of our study lies in the integrated assessment of MDRO colonisation and cUTIs within the same hospitalisation episode, allowing a clinically contextualised interpretation of their relationship in hospitalised KT recipients.

Among admissions with cUTI, longer LoS remained associated with urinary stent presence and BSI after multivariable adjustment.

These findings reinforce that cUTIs in KT recipients, particularly when accompanied by BSI, are associated with a more complex clinical course and greater resource utilisation.

From a clinical standpoint, they also emphasise the potential value of optimising device management strategies and early risk stratification for severe courses.

In the context of KT, several risk factors for UTI occurrence and severity have been identified [2,8,10,11,33]. In particular, routine urinary stent positioning reduces the risk of urological complications, but prolonged maintenance increases the risk of urinary tract infections [2,34,35].

Our findings suggest that urinary stents may contribute to cUTI occurrence and are associated with prolonged LoS in KT patients; however, their association with cUTI did not reach statistical significance in multivariable analysis.

A key finding of our study is the identification of a potential clinical link between colonisation and infection.

MDRO rectal carriage was common (43.1%), particularly in cUTI (53.7%), and was driven by CP-KP carriage (31.7%). Although this association did not retain statistical significance in multivariable models, likely due to limited power, rectal CP-KP colonisation showed a consistent relationship with cUTI aetiology, with a PPV of 75% and an NPV of 86.4%. However, due to the retrospective design and the timing of rectal screening, a clear temporal relationship between colonisation and infection cannot be definitively established. Consistent with these findings, a recent analysis revealed that MDRO colonisation, particularly CRE colonisation, significantly increases the likelihood of infections caused by the same colonising MDROs in SOTRs [32]. Furthermore, a strong correlation between CRE colonisation and CRE BSIs has been consistently demonstrated in liver transplant settings [13,36,37].

These data suggest that, in endemic settings, rectal screening may contribute to risk stratification of hospitalised KT recipients with suspected cUTI; however, its role in guiding empirical therapy was not directly evaluated in this study and should therefore be considered exploratory.

Finally, the results of our study highlight that, although VRE/VVE rectal colonisation is present (24.6%), it did not show a significant association with cUTIs in our cohort; however, this finding should be interpreted cautiously, as it may reflect limited statistical power rather than a true lack of clinical relevance [38,39].

From a stewardship and clinical management perspective, our findings suggest that a “precision empiric” approach integrating local epidemiology, device-related risk factors, and colonisation status may help inform empirical therapy; however, this approach should be considered hypothesis-generating and requires prospective validation.

Such an approach may be relevant in the current therapeutic landscape, where multiple agents are available for CRE [40]. Nonetheless, their optimal use still depends on early recognition of resistance mechanisms and careful antimicrobial stewardship to preserve efficacy and minimise toxicity [25,41].

This study has several limitations. First, its retrospective and single-centre design may limit the generalisability of the findings to other settings with different epidemiological characteristics. Second, the relatively small sample size may have reduced the statistical power to detect significant associations and contributed to wide confidence intervals and limited model stability. Third, although all consecutive admissions were included, residual confounding cannot be excluded. Finally, the observational design precludes any causal inference, and the findings should be interpreted as exploratory and hypothesis-generating.

In addition, the retrospective nature of the study introduces the possibility of residual confounding, as not all potential determinants of infection risk and clinical outcomes could be systematically captured (e.g., antibiotic exposure within 90 days before hospitalisation). Clinical outcomes were limited to length of stay and bloodstream infection, as data on other transplant-specific outcomes such as graft loss, rejection, renal function deterioration, readmission, and mortality were not consistently available.

Furthermore, detailed information on antimicrobial exposure, including prior treatments and the appropriateness of empirical therapy, was not consistently available and therefore could not be included in the analysis. Similarly, other clinically relevant factors, such as infection severity at admission, ICU admission, graft dysfunction, and timing of source control, were not systematically recorded and were not incorporated into the model.

## 4. Materials and Methods

### 4.1. Study Design and Population

We conducted a single-centre retrospective observational study at the National Institute for Infectious Diseases ‘Lazzaro Spallanzani’ IRCCS (Rome, Italy). All consecutive kidney or kidney–pancreas transplant recipients admitted for infectious diseases between 30 June 2023 and 31 January 2025 were included. Each hospitalisation was analysed as a separate event.

Potential bias due to the retrospective design was minimised by including all consecutive eligible admissions during the study period and by using standardised microbiological methods.

This study is reported in accordance with the Strengthening the Reporting of Observational studies in Epidemiology (STROBE) statement for retrospective observational studies (Supplementary Table S2).

### 4.2. Data and Definitions

Demographic, clinical, biochemical, microbiological and treatment data (e.g., immunosuppression and antimicrobials) were extracted from clinical records.

cUTI (pyelonephritis or upper tract UTI) was defined by the presence of at least one of the following clinical features: fever, chills, malaise, haemodynamic instability, leukocytosis (without another apparent aetiology), flank/allograft pain, or bacteraemia caused by the same organism identified in urine, together with leukocyturia (>10 white blood cells (WBC)/mL) and significant bacteriuria (≥10^5^ CFU/mL in clean-catch midstream urine or ≥10^4^ CFU/mL in catheter-derived urine) [1].

Chronic kidney disease (CKD) was defined according to the KDIGO as abnormalities of kidney structure or function present for >3 months, indicated by estimated glomerular filtration rate (eGFR) <60 mL/min/1.73 m^2^ or markers of kidney damage; eGFR was calculated using the CKD-EPI equation [17].

### 4.3. Microbiology

Urine cultures were systematically obtained, while blood cultures were performed based on clinical indication (e.g., fever, haemodynamic instability, or suspected sepsis) and were not routinely collected in all cUTI admissions. Whenever indicated, cultures were obtained prior to antibiotic administration. Isolates from urine, blood and rectal samples were identified by MALDI-TOF Byotiper Sirius System (Bruker Daltonics, Bremen, Germany) and MBT Compass software (v4.2) and antimicrobial susceptibility testing was performed using the Phoenix system (Becton Dickinson, Franklin Lakes, NJ, USA), interpreted according to the European Committee on Antimicrobial Susceptibility Testing (EUCAST) criteria (v15.0) [42]. Polymicrobial cultures were interpreted according to clinical and microbiological context, and findings considered as likely contamination were excluded from analysis.

### 4.4. Rectal Screening

As per institutional protocol, rectal screening for CRE and VRE/VVE was performed within 48 h of admission and then weekly during hospitalisation. For the purpose of the analysis, only rectal swab results obtained at admission (within 48 h) were considered for evaluating the association between colonisation and infection. Rectal swabs were tested using a multiplex real-time PCR assay (Allplex™ Entero-DR, Seegene, Seoul, Republic of Korea) targeting carbapenemase genes (*bla*_VIM_, *bla*_NDM_, *bla*_OXA-48_, *bla*_KPC_, *bla*_IMP_) and *van* genes (*van*A, *van*B). PCR-positive samples were cultured on selective chromogenic media for organism isolation and susceptibility testing. Culture confirmation of PCR-positive samples was performed for microbiological characterization only, and concordance between PCR and culture results was not formally evaluated.

### 4.5. Statistical Analysis

Categorical variables were expressed as absolute values and percentages, while the quantitative ones were reported as medians and IQRs. Comparison between groups was performed by the chi-square test or Fisher’s exact test (as appropriate) for categorical variables and the nonparametric Wilcoxon rank-sum test for continuous variables.

A multivariable logistic regression model was used to explore predictors of cUTI as compared with other infections. Since repeated observations from the same subject were possible, intra-subject correlation was accounted for by estimating cluster-robust standard errors at the patient level. Given the limited sample size and the relatively large number of candidate predictors, variable selection was performed using a forward stepwise logistic regression procedure with an entry criterion of *p* = 0.10. This procedure was used as an exploratory strategy to obtain a parsimonious model and reduce the risk of overparameterization. To mitigate the instability inherent in stepwise selection, additional model-selection checks were performed by comparing candidate parsimonious models using the BIC. In accordance with the commonly used rule of thumb of approximately 10 events per estimated parameter, the maximum number of parameters allowed in the final model was defined based on the number of observations in the less frequent outcome category. Within this constraint, we evaluated whether the predictors selected through the stepwise procedure were consistent with those retained in the model with the lowest BIC. The final set of predictors was further assessed for clinical plausibility.

Among patients with cUTI, variables associated with LoS were assessed using median regression. Quantile regression with clustered standard errors was performed using the Stata qreg2 command and regression coefficients were reported together with 95% CIs and *p*-values. To build a parsimonious multivariable model, only variables associated with LoS at *p* < 0.15 in univariable analysis were considered for inclusion.

For patients in whom the aetiological agent was identified, the relationship between cUTI due to a specific pathogen and rectal colonisation with the same pathogen was assessed by calculating the PPV and NPV of the rectal swab.

A two-sided test with a *p*-value less than 0.05 was considered statistically significant. Analyses were conducted with Stata Software (Stata Corp. 2021. Stata Statistical Software: Release 17. Stata Corp LLC, College Station, TX, USA). Missing data were negligible in this dataset, with the exception of the aetiological agent in 7 cUTI cases; therefore, analyses were effectively conducted on complete cases. No formal subgroup or interaction analyses were performed, and no sensitivity analyses were conducted.

## 5. Conclusions

Our study highlights the burden of cUTIs caused by MDROs in KT recipients and underscores the potential relevance of rectal CP-KP colonisation in this context.

These findings should be interpreted with caution given the retrospective design and limited sample size. Larger, prospective, multi-centre studies are needed to confirm these associations and to clarify whether colonisation status may contribute to risk stratification and clinical decision-making in this setting.

## Figures and Tables

**Table 1:** Characteristics at the baseline of hospital admissions among kidney transplanted (KT) patients admitted for complicated urinary tract infections (UTIs) compared with admissions for other infections.

Characteristics	Infections at Hospital Admission		Total	*p*
	UTIs	Other Infection		
**Overall**, *n* (%)	41 (63.1)	24 (36.9)	65 (100)	
**Age**, median (IQR)	56 (48–64)	61 (42–68)	57 (48–67)	0.812
**Sex**, *n* (%)				0.487
Female	19 (46.3)	9 (37.5)	28 (43.1)	
Male	22 (53.7)	15 (62.5)	37 (56.9)	
**Comorbidities**, *n* (%)				
Diabetes	7 (17.1)	2 (8.3)	9 (13.8)	0.466
Arterial hypertension	31 (75.6)	22 (91.7)	53 (81.5)	0.184
**eGFR** mL/min/1.73 m^2^, median (IQR)	33 (18–50)	28 (20–44)	30 (18–47)	0.605
**CKD**				0.840
≥60	7 (17.1)	3 (12.5)	10 (15.4)	
30–59	15 (36.6)	7 (29.2)	22 (33.8)	
15–29	13 (31.7)	9 (37.5)	22 (33.8)	
<15	6 (14.6)	5 (20.8)	11 (16.9)	
**Type of transplant**, *n* (%)				0.733
Kidney	34 (82.9)	21 (87.5)	55 (84.6)	
Kidney and Pancreas	7 (17.1)	3 (12.5)	10 (15.4)	
**Years from transplant**, *n* (%)				0.091
<1 year	16 (39.0)	4 (16.7)	20 (30.8)	
1 to 5 years	11 (26.8)	12 (50.0)	23 (35.4)	
>5 years	14 (34.1)	8 (33.3)	22 (33.8)	
**Stent**, *n* (%)				0.016
No	21 (51.2)	20 (83.3)	41 (63.1)	
Yes	20 (48.8)	4 (16.7)	24 (36.9)	
**Immunosuppressive therapy**, *n* (%)				
Mycophenolate mofetil	30 (73.2)	17 (70.8)	47 (72.3)	0.839
Corticosteroid	41 (100)	21 (87.5)	62 (95.4)	0.046
Corticosteroid dose				
<10 mg/day	24 (58.5)	16 (76.2)	40 (64.5)	0.262
≥10 mg/day	17 (41.5)	5 (23.8)	22 (35.5)	
**MDROs Rectal swab**, *n* (%)	22 (53.7)	6 (25.0)	28 (43.1)	0.024
**Total *Enterococcus faecium* VRE/VVE**	12 (29.3)	4 (16.7)	16 (24.6)	0.373
**Total CP-KP**	13 (31.7)	1 (4.2)	14 (21.5)	0.011
**BSI**, *n* (%)	9 (22.0)	4 (16.7)	13 (20.0)	0.753
**UTIs with identified agent**, *n* (%)	34 (82.9)			
**Total *Escherichia coli***	14 (41.2)			
**Total CP-KP**	12 (35.3)			

**Abbreviations:** IQR, interquartile range; eGFR, estimated glomerular filtration rate; CKD, chronic kidney disease; MDROs, multidrug-resistant organisms; BSI, bloodstream infection; VRE, Vancomycin-Resistant enterococci; VVE, Vancomycin-Variable enterococci; CP-KP, carbapenemase-producing *Klebsiella pneumoniae* (CP: KPC, NDM, and OXA-48).

**Table 2:** Association between CP-KP rectal colonisation and CP-KP urinary tract infection, positive and negative predictive values among 34 cUTIs with identified agent.

CP-KP cUTI	CP-KP Rectal Swab		Total	*p*
	No, *n* (%)	Yes, *n* (%)	*n* (%)	
**No**	19 (86.4)	3 (25.0)	22 (64.7)	0.001
**Yes**	3 (13.6)	9 (75.0)	12 (35.3)	
PPV of rectal swab: 75.0% (95% CI 42.8–94.5%) NPV of rectal swab: 86.4% (95% CI 65.1–97.1%)				

**Abbreviations:** CP-KP, carbapenemase-producing *Klebsiella pneumoniae* (CP: KPC, NDM, and OXA-48); cUTI, complicated urinary tract infection; CI, confidence interval; PPV, positive predictive value; NPV, negative predictive value.

**Table 3:** Univariable and multivariable analysis of factors associated with length of hospital stay (LoS) in 41 admissions of kidney and kidney/pancreas transplanted patients with complicated urinary tract infections (cUTIs).

Characteristics	Quantile Regression			
	Univariable		Multivariable *	
	Coefficient (95% CI)	*p*	Coefficient (95% CI)	*p*
**Age in years** (for a 10-year increment)	2.0 (−0.5; 4.5)	0.112	2.3 (0.7; 4.0)	0.007
**Male vs. Female**	3.0 (−4.2; 10.2)	0.405		
**Comorbidities**, presence vs. absence				
Diabetes	−4.0 (−12.2; 4.2)	0.332		
Arterial hypertension	0.0 (−8.0; 8.0)	1.000		
**eGFR** mL/min/1.73 m^2^	−0.04 (−0.2; 0.1)	0.528		
**CKD**				
≥60	Reference			
30–59	−5.0 (−11.4; 1.4)	0.122		
15–29	3.0 (−6.1; 12.1)	0.509		
<15	4.0 (−5.5; 13.5)	0.400		
**Type of transplant**, Kidney and Pancreas vs. Kidney	−4.0 (−11.7; 3.7)	0.298		
**Years from transplant**	−0.3 (−0.7; 0.1)	0.164		
<1 year	Reference			
1 to 5 years	−5.0 (−16.3; 6.3)	0.374		
>5 years	−6.0 (−17.8; 5.8)	0.308		
**Urinary stent**, presence vs. absence	7.0 (−1.5; 15.5)	0.104	6.8 (1.5; 12.2)	0.014
**Immuno-suppressive therapy**				
Mycophenolate Mofetil, yes vs. no	−1.0 (−9.0; 7.0)	0.802		
**Corticosteroid dose**				
<10 mg/day	Reference			
≥10 mg/day	0.0 (−6.9; 6.9)	1.000		
**UTIs with identified agent**, yes vs. no	−4.0 (−12.6; 4.6)	0.354		
**Agent: *Escherichia coli***, yes vs. no	−2.0 (−10.4; 6.4)	0.634		
**Agent: CP-KP**, yes vs. no	7.0 (−4.3; 18.3)	0.218		
**MDROs Rectal swab at admission**, yes vs. no	−4.0 (−13.0; 5.0)	0.372		
**Agent: *E. faecium* VRE/VVE**, yes vs. no	4.0 (−16.8; 24.8)	0.700		
**Rectal colonisation or urinary infection with CP-KP**, yes vs. no	2.0 (−5.2; 9.2)	0.579		
**BSI**, yes vs. no	14.0 (5.7; 22.3)	0.002	12.2 (0.8; 23.6)	0.037

* Variables included in the model were selected among those with a *p*-value < 0.15 in the univariable analysis, specifically age, urinary stent, and BSI. **Abbreviations**: CI, confidence interval; eGFR, estimated glomerular filtration rate; UTI, urinary tract infection; cUTI, complicated urinary tract infections; CKD, chronic kidney disease; CP-KP, carbapenemase-producing *Klebsiella pneumoniae*; BSI, bloodstream infection; MDROs, multidrug-resistant organisms; VRE, vancomycin-resistant enterococci; VVE, vancomycin-variable enterococci.

## Data Availability

The datasets generated and/or analyzed during the current study are archived at the authors’ institution and are available from the corresponding author upon reasonable request.

## Supplementary Materials

The following supporting information can be downloaded at https://www.mdpi.com/article/10.3390/antibiotics15070656/s1, Table S1: Overview of the microbiological characteristics of MDRO rectal swabs, bloodstream infections (BSIs), and cUTI aetiological agents according to type of infection at hospital admission; Table S2: The “Strengthening the Reporting of Observational studies in Epidemiology” (STROBE) statement for retrospective observational studies.

## Abbreviations

The following abbreviations are used in this manuscript:

UTIs	Urinary tract infections
KT	Kidney transplant
BSI	Bloodstream infection
MDRO	Multidrug-resistant organism
ESBL	Extended-spectrum β-lactamase
CRE	Carbapenem-resistant *Enterobacterales*
VRE	Vancomycin-resistant enterococci
VVE	Vancomycin-variable enterococci
cUTI	Complicated urinary tract infection
CP-KP	Carbapenemase-producing *Klebsiella pneumoniae*
LoS	Length of hospital stay
KDIGO	Kidney Disease: Improving Global Outcomes
eGFR	estimated glomerular filtration rate
IQR	Interquartile range
CKD	Chronic kidney disease
BIC	Bayesian Information Criterion
ICU	Intensive Care Unit
OR	Odds ratio
CI	Confidence interval
PPV	Positive predictive value
NPV	Negative predictive value
WBC	White blood cells
SOTRs	Solid organ transplant recipients
STROBE	Strengthening the Reporting of Observational Studies in Epidemiology
CR-KP	Carbapenem-resistant *K. pneumoniae*
EUCAST	European Committee on Antimicrobial Susceptibility Testing
