# Supplementary material for: Gut Colonisation and Multidrug-Resistant Urinary Tract Infections in Hospitalised Kidney Transplant Recipients: A Single-Centre Retrospective Study
Source: Antibiotics (Basel). 2026 Jul 1;15(7):656. doi: 10.3390/antibiotics15070656 (PMC13405800; doi:10.3390/antibiotics15070656)
Supplement: Supplementary file 1 [file antibiotics-15-00656-s001.zip › Table S1.pdf]

Table S1. Overview of the microbiological characteristics of MDRO rectal swabs, bloodstream infections (BSIs), and cUTI aetiological agents according to type of infection at hospital admission.

Characteristics	Infections at hospital admission		Total
	**cUTIs**, n (%)	**Other infection**, n (%)	
**Overall**	41 (63.1)	24 (36.9)	65 (100)
**BSI identified agent**	9 (22.0)	4 (16.7)	13 (20.0)
Escherichia coli	3 (7.3)	0 (0)	3 (4.6)
Klebsiella aerogenes ESBL	0 (0)	1 (4.2)	1 (1.5)
Klebsiella pneumoniae CTX-M	1 (2.4)	0 (0)	1 (1.5)
Klebsiella pneumoniae ESBL	1 (2.4)	0 (0)	1 (1.5)
Klebsiella pneumoniae KPC	3 (7.3)	0 (0)	3 (4.6)
Paenibacillus spp.	0 (0)	1 (4.2)	1 (1.5)
Staphylococcus aureus	0 (0)	2 (8.3)	2 (3.1)
Stenotrophomonas maltophilia	1 (2.4)	0 (0)	1 (1.5)
**MDRO Rectal swab identified agent**	22 (53.7)	6 (25.0)	28 (43.1)
Enterococcus faecalis VRE	0 (0)	1 (4.2)	1 (1.5)
Enterococcus faecium VRE	8 (19.5)	4 (16.7)	12 (18.5)
Enterococcus faecium VRE/Klebsiella pneumoniae KPC	2 (4.9)	0 (0)	2 (3.1)
Enterococcus faecium VVE	1 (2.4)	0 (0)	1 (1.5)
Enterococcus faecium VVE/Klebsiella pneumoniae KPC	1 (2.4)	0 (0)	1 (1.5)
Klebsiella pneumoniae KPC	7 (17.1)	1 (4.2)	8 (12.3)
Klebsiella pneumoniae NDM	2 (4.9)	0 (0)	2 (3.1)
Klebsiella pneumoniae OXA-48	1 (2.4)	0 (0)	1 (1.5)
**UTIs identified agent**	34 (82.9)		
Escherichia coli	4 (11.8)	------	
Escherichia coli ESBL	7 (20.6)	------	
Klebsiella pneumoniae	1 (2.9)	------	
Klebsiella pneumoniae ESBL	6 (17.6)	------	
Klebsiella pneumoniae KPC	9 (26.5)	------	
Klebsiella pneumoniae NDM/*E. coli*	2 (5.9)	------	
Klebsiella pneumoniae OXA-48	1 (2.9)	------	
Klebsiella pneumoniae/E. coli	1 (2.9)	------	
Pseudomonas aeruginosa	1 (2.9)	------	
Enterococcus faecium VS/VVE	2 (5.9)	------	

Abbreviations: MDRO, multidrug-resistant organisms; cUTIs, complicated urinary tract infections; BSI, bloodstream infection.
