# Supplementary material for: Gut Colonisation and Multidrug-Resistant Urinary Tract Infections in Hospitalised Kidney Transplant Recipients: A Single-Centre Retrospective Study
Source: Antibiotics (Basel). 2026 Jul 1;15(7):656. doi: 10.3390/antibiotics15070656 (PMC13405800; doi:10.3390/antibiotics15070656)
Supplement: Supplementary file 1 [file antibiotics-15-00656-s001.zip › Table S2.pdf]

Table S2. The "Strengthening the Reporting of Observational studies in Epidemiology" (STROBE) statement for retrospective observational studies.

	Item No	Recommendation
**Title and abstract**	1	(*a*) single-centre retrospective study
		(*b*) Retrospective single-centre observational study of consecutive kidney or kidney–pancreas transplant recipients hospitalised for infectious diseases between 30 June 2023 and 31 January 2025. Abstract-Methods
**Introduction**		
Background/rationale	2	Background explains clinical importance of UTIs after kidney transplant and rising MDROs; rationale for assessing rectal colonisation as predictor of cUTI aetiology. Introduction
Objectives	3	Primary objective: assess burden and microbiology of complicated UTIs among hospitalised kidney transplant recipients. Secondary objective: evaluate whether rectal MDRO colonisation predicts cUTI aetiology and outcomes, focusing on CP-KP. Introduction
**Methods**		
Study design	4	Retrospective single-centre observational study; each hospitalisation analysed as separate event. Methods
Setting	5	Setting: tertiary infectious diseases institute (INMI L. Spallanzani), Rome, Italy; period 30 June 2023–31 January 2025; hospitalised kidney and kidney–pancreas transplant recipients.
Participants	6	(*a*) Inclusion: all consecutive kidney or kidney–pancreas transplant recipients admitted for infectious diseases during study period; each hospitalisation treated as separate event; n=65 hospitalisations, 52 patients. Methods
		(*b*) For matched studies, give matching criteria and number of exposed and unexposed
Variables	7	UTI ($\geq 10^5$ CFU/mL clean-catch or $\geq 10^4$ CFU/mL catheter urine + symptoms + leukocyturia); cUTI and BSI defined clinically and microbiologically; CKD per KDIGO; rectal colonisation defined by PCR and culture. Methods
Data sources/ measurement	8*	Data from clinical records; urine and blood cultures obtained before antibiotics when possible; identification by MALDI-TOF; AST by Phoenix; rectal PCR Allplex™ Entero-DR and culture on selective media. Methods
Bias	9	Potential selection bias minimised by including all consecutive admissions; standardised microbiological methods used to reduce measurement bias; limitation: retrospective design and single-centre setting.Methods and Conclusion
Study size	10	Study size determined by consecutive admissions during study period (65 hospitalisations); no a priori sample size calculation (exploratory/observational study). Methods and Results
Quantitative variables	11	Continuous variables reported as medians and IQR; categorical as counts and percentages; eGFR calculated by CKD-EPI. Methods
Statistical methods	12	(*a*) Chi-square/Fisher for categorical; Wilcoxon rank-sum for continuous; stepwise logistic regression (forward, entry p=0.100) for factors associated with cUTI; quantile regression (median) for LoS; models account for repeated hospitalisations; analyses with Stata 17 (qreg2). Methods
		(*b*) No formal subgroup or interaction analyses were performed. Methods
		(*c*) The rare occurrence of missing data was addressed through complete-case analysis. Methods
		(*d*) N/A

		(*e*) Not performed
**Results**		
Participants	13*	(a) 65 hospitalisations included; 52 unique patients; 7 patients with 2 admissions, 3 with 3 admissions. Results
		(b) N/A
		(c) Not included
Descriptive data	14*	(a) Baseline characteristics summarised in Table 1 (age, sex, comorbidities, eGFR, stent presence, immunosuppression).
		(b) 52 patients
		(c) N/A due to the cross-sectional analysis of hospitalisations, which does not include longitudinal follow-up beyond admission.
Outcome data	15*	Number of cUTIs (41/65), BSI among cUTIs (22% of cUTIs), pathogens identified in 34/41 cUTIs; *K. pneumoniae* and *E. coli* frequencies; resistance patterns (ESBL, carbapenemases). Results, Table 1 and Table 2
Main results	16	(*a*) Unadjusted associations were evaluated using bivariate comparisons with chi-square/Fisher's exact and Wilcoxon rank-sum tests (Table 1), and for length of stay (LoS) with univariable median quantile regression (Table 3). Adjusted analyses accounted for repeated hospitalisations. In the multivariable logistic regression for factors associated with cUTI (covariates selected using a forward stepwise procedure with an entry *p*-value of 0.10), the main independent factors were urinary stent presence (odds ratio (OR) 3.3, 95% confidence interval (CI) 0.8–13.1) and CP-KP rectal colonisation (OR 7.1, 95% CI 0.7–66.2). In the multivariable median quantile regression analysis of LoS among cUTIs (covariates selected from the univariable analysis), bloodstream infection (BSI) increased LoS by 12.0 days (95% CI 0.8–23.6), urinary stents by 6.8 days (95% CI 1.5–12.2) and age by 2.3 days per 10 years (95% CI 0.7–4.0).
		(*b*) Category boundaries: eGFR categories were defined as ≥60, 30–59, 15–29, and <15 mL/min/1.73m²; time from transplant categories were <1 year, 1–5 years, and >5 years (Table 1)
		(*c*) N/A
Other analyses	17	PPV/NPV for CP-KP colonisation vs infection; analyses of LoS by BSI, stent, age; no formal sensitivity analyses performed. Results, Table 2 and Supplementary table 1
**Discussion**		
Key results	18	cUTIs were the leading cause of admission; Gram-negative pathogens predominated; rectal CP-KP colonisation associated with CP-KP cUTI (PPV 75%, NPV 86.4%); MDRO colonisation more frequent in cUTI vs other infections. Discussion
Limitations	19	Retrospective single-centre design limits generalisability; rectal screening at admission prevents establishing temporal sequence in all cases; limited sample size for some subgroup analyses. Discussion
Interpretation	20	Interpretation consistent with objectives and literature: colonisation-informed empirical therapy may be useful in endemic settings; results hypothesis-generating and require prospective validation.
Generalisability	21	External validity limited by single-centre setting and hospitalised population; findings most applicable to centres with similar MDRO epidemiology.

Other information		
Funding	22	Funding sources: Italian Ministry of Health through Ricerca Corrente linea 3, project Italian Ministry of Health through Ricerca Corrente-Linea 2, Project 5 and Ricerca Corrente – Linea 3, Project 2; conflicts of interest to be declared.

*Give information separately for exposed and unexposed groups.

Note: An Explanation and Elaboration article discusses each checklist item and gives methodological background and published examples of transparent reporting. The STROBE checklist is best used in conjunction with this article (freely available on the Web sites of PLoS Medicine at http://www.plosmedicine.org/, Annals of Internal Medicine at http://www.annals.org/, and Epidemiology at http://www.epidem.com/). Information on the STROBE Initiative is available at http://www.strobe-statement.org.
